# Gene-pseudogene evolution: a probabilistic approach

**DOI:** 10.1186/1471-2164-16-S10-S12

**Published:** 2015-10-02

**Authors:** Owais Mahmudi, Bengt Sennblad, Lars Arvestad, Katja Nowick, Jens Lagergren

**Affiliations:** 1 Department of Clinical Microbiology, Karolinska University Hospital, Stockholm, Sweden; 2 Artherosclerosis research unit, Department of Medicine, Karolinska Institute, Science for Life Laboratory, Stockholm, Sweden; 3 Department of Numerical Analysis and Computer Science, Swedish e-Science Research Centre, Stockholm University, Stockholm, Sweden; 4 TFome Research Group, Bioinformatics Group, Interdisciplinary Center of Bioinformatics, Department of Computer Science, University of Leipzig, Härtelstrasse 16-18, D-04107 Leipzig, Paul-Flechsig-Institute for Brain Research, University of Leipzig, Jahnallee 59, D-04109 Leipzig, Germany

**Keywords:** pseudogene evolution, probabilistic model, birth-death model, gene tree, species tree, sequence evolution, zinc fingers, olfactory receptors, gene family

## Abstract

Over the last decade, methods have been developed for the reconstruction of gene trees that take into account the species tree. Many of these methods have been based on the probabilistic duplication-loss model, which describes how a gene-tree evolves over a species-tree with respect to duplication and losses, as well as extension of this model, e.g., the DLRS (Duplication, Loss, Rate and Sequence evolution) model that also includes sequence evolution under relaxed molecular clock. A disjoint, almost as recent, and very important line of research has been focused on non protein-coding, but yet, functional DNA. For instance, DNA sequences being pseudogenes in the sense that they are not translated, may still be transcribed and the thereby produced RNA may be functional.

We extend the DLRS model by including pseudogenization events and devise an MCMC framework for analyzing extended gene families consisting of genes and pseudogenes with respect to this model, i.e., reconstructing gene-trees and identifying pseudogenization events in the reconstructed gene-trees. By applying the MCMC framework to biologically realistic synthetic data, we show that gene-trees as well as pseudogenization points can be inferred well. We also apply our MCMC framework to extended gene families belonging to the Olfactory Receptor and Zinc Finger superfamilies. The analysis indicate that both these super families contains very old pseudogenes, perhaps so old that it is reasonable to suspect that some are functional. In our analysis, the sub families of the Olfactory Receptors contains only lineage specific pseudogenes, while the sub families of the Zinc Fingers contains pseudogene lineages common to several species.

## Introduction

The human genome probably contains almost as many pseudogenes as protein-coding genes, since the number of predicted pseudogenes ranges from 10,000 to 20,000 [1], Pseudogenes were initially thought to be nonfunctional genes and often termed as *junk DNA*. Jacq and his colleagues used the term pseudogene for the first time, when they discovered a version of the gene coding for 5S rRNA that was truncated but retained the homology with the active gene in Xenopus laevis [2]. Pseudogenes have earlier been defined as defunct copies of genes that have lost their potential as DNA templates for functional protein products [3] and they have been considered to be *genomic fossils*, evolving without selective pressure. More recently, it has been observed that some pseudogenes are more conserved. Direct evidence of functionality has also been reported for some pseudogenes.

A gene may get pseudogenized by acquiring a spontaneous mutation preventing either transcription or meaningful translation, e.g., due to a frame shift or introduction of a premature stop codons, of the gene, thus forming a *unitary pseudogene *[4]. Pseudogenization is one of the possible fates of a duplicated gene leading to a *duplicated pseudogene*. A *processed pseudogene*, the result of integration of an mRNA into the genome by reverse transcription, is typically "dead on arrival", since integration close to a promoter is a necessary requirement for their transcriptional activity that most often is not satisfied [1], In the case of humans, the retro-transposition of the mRNA appears to be mediated by long interspersed nuclear element (LI) [5].

Pseudogenes are present in a wide range of species, including plants [6], prokaryotes [7], insects [8], nematode worms [9], but they are particularly numerous in mammals [1]. A pseudogene without any function should evolve neutrally, i.e., evolve free of evolutionary pressure and follow random drift. However, recent studies have challenged this view and found that some pseudogenes are not only conserved, but they also have some potential function. For instance, it was found that in Drosophila Est-6 pseudogene synonymous mutations were far more frequent than non-synonymous mutations [10]. In some of the pseudogenes present in chicken, i.e. IglV and IghV, and in mouse i.e. VH, the number of stop codons in the coding sequence region is far lower than expected under neutral evolution [11,12]. It has also been observed that some pseudogenes retain conservation across species, for example, during the analysis of major histocompatibility complex extended class II, two pseudogenes were found to be homologous to human HIV TAT-specific factor-1-like and zinc finger like pseudogenes [13]. Another study showed that a transcribed region of pseudogene Makorin1-1p exhibits rates of point and indel substitutions that are two to four times lower than those in untranscribed region, suggesting functional constraints on the transcribed region [14]. However, evidence of the Makorin1-1p being non-functional has been provided by Gray et al. [15]. Further, in a genome-wide survey of pseudogenes, Svensson et al. [16] identified ancient pseudogenes common to human and mouse, that originated by a duplication before the speciation split that were highly conserved. A comparison of transcribed human pseudogenes with rhesus monkey showed that 50% of the pseudogenes are conserved with rhesus monkey, and 3% are conserved even with mouse [17]. Marques et al. [18] considered 48 rodent specific pseudogenes that lost their protein coding ability during the rodent evolution and a substantial fraction of these pseudogenes are still expressed despite lacking an apparent open reading frame. It is, thus, important to understand how and when the specific pseudogenes have been formed, and how they evolved in extended gene families, consisting of genes and pseudogenes.

Goodman et al. [19] introduced a parsimony based concept of reconciliation between a gene-tree and a species-tree, which explains possible incongruences between the two trees in terms of duplications and losses. Parsimony based reconciliation has attracted a lot of attention, and a wealth of methods have been developed following Goodman et al. Arvestad et al. [20] extended this line of research by introducing Duplication-Loss model (DL), the first probabilistic model of how a gene-tree evolves inside a species-tree, with respect to gene duplications and losses, and showed how to simultaneously reconstruct a gene-tree and reconcile it with the species-tree under the DLRS model, which also includes rate variation and sequence evolution [20-22]. In order to facilitate proper analysis of gene families including pseudogenes, i.e., gene-tree reconstruction and identification of pseudogenization events, we extend this model by introducing the possibility of pseudogenization of gene lineages. The resulting model, hence, integrates the evolution of genes and pseudogenes that may undergo duplication/loss events, gene-to-pseudogene conversions, and sequence evolution under a relaxed molecular clock for substitution rates. We devise MCMC based methods that allows data analysis with respect to this model, and apply it on synthetic as well as biological datasets. The biological datasets consists of genes and pseudogenes from two largest gene families in vertebrates, i.e. Olfactory Receptors and Zinc Fingers. Olfactory receptors are studied across human, dog, opossum, and platypus, while zinc fingers are studied across the four primate species human, chimpanzee, rhesus monkey, and orangutan.

## Methods

In this section we first introduce the Pseudogenization, Duplication, Loss, Rate and Sequence evolution model, PDLRS. We start by first defining some basic terms. A species-tree is a rooted binary tree that represents evolutionary history of species where leaves represent extant species and internal vertices represent speciation events. A gene-tree is also a rooted binary tree that represents evolutionary history of a set of genes. A gene-tree may have genes or pseudogenes as its leaves.

### The PDLRS model

The PDLRS model is an extension of the DLRS model obtained by also including pseudogenization events. The model describes how a gene lineage evolves inside a species-tree with a degree one root, by starting at the root and subsequently evolving towards the leaves while being exposed to gene duplication, gene loss, and pseudogenization events at rates *δ*, μ, and ψ, respectively. Also, when a gene lineage reaches a species-tree vertex, it always (i.e., deterministically) bifurcates and the two so contained gene lineages continue to evolve below the species-tree vertex, one in each of its two outgoing species-tree edges.

**Figure 1:**
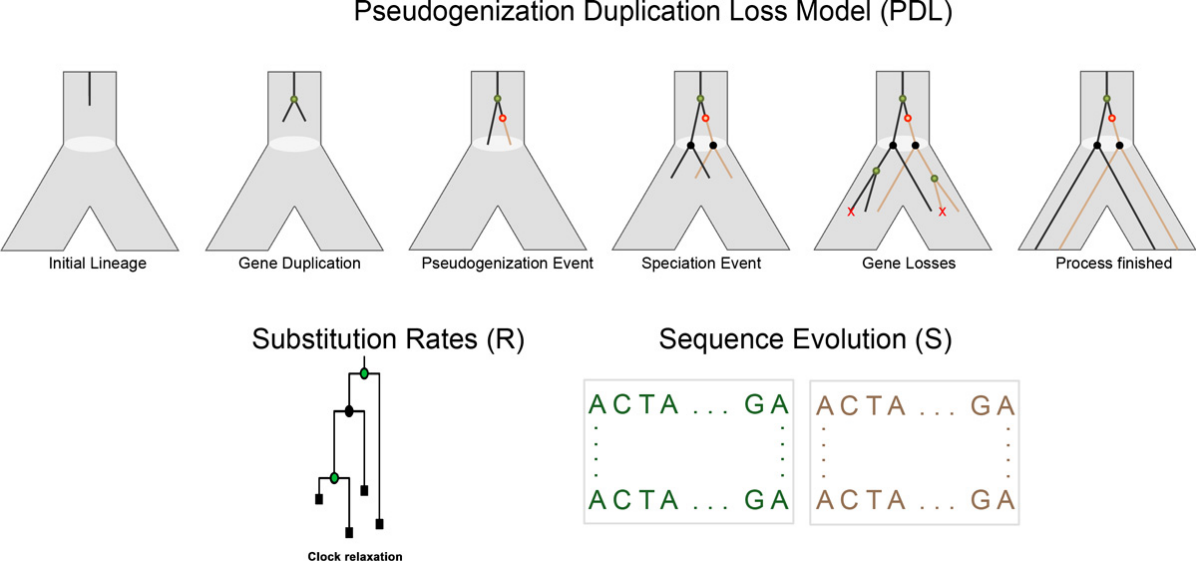
**Pseudogenization, Duplication, Loss, Sequence evolution & Rates (PDLRS)**. Evolution of a gene and pseudogene lineages inside a species tree edge is modelled by a birth-death process. A gene/pseudogene lineage may come across a duplication event, or a speciation event. A gene lineage (represented by black lineages) may convert into a pseudogene lineage (represented by brown lineages). Every time a gene/pseudogene lineage passes through a speciation event, it splits into two independent gene lineages. A gene lineage may also be lost. After pruning all lost lineages, the final gene tree is obtained. A relaxed molecular clock is employed to obtain branch lengths. Finally, a standard sequence evolution model generates sequences over the gene tree with branch lengths. Green and brown colors represents gene and pseudogene sequence evolution, respectively.

Although during this process, a gene lineage may switch into a pseudogene lineage, a pseudogene lineage is not allowed to switch back to a gene lineage. Pseudogenization events introduce degree two vertices in the gene-tree. A pseudogene lineage otherwise behaves as a gene lineage, it may duplicate or become lost during the evolution, and it deterministically bifurcates when it reaches a species-tree vertex. A lineage that reaches the leaves of the species-tree gives rise to a leaf in the gene-tree, representing an extant gene or pseudogene. Vertices and edges of the gene-tree that do not lead to any such extant leaves are, however, pruned from the gene-tree (Figure 1). Since this process takes place in a species-tree with time on its vertices and edges, each event occurs at a specific time. Whenever an event creates a new gene-tree vertex the time of the event is associated with the new vertex.

In order to obtain a relaxed molecular clock, rates are sampled independently from a Γ-distribution (parameterized by a mean and a variance) for each edge, and an edge with time *t *and rate r is assigned a length *l*. Finally, sequences are evolved over this gene-tree with its lengths. Recall that pseudogenization events introduce degree two vertices in the gene-tree. Over an edge where the parental vertex is a gene a model of sequence evolution suitable for genes is used, while when the parental vertex represent a pseudogene (and, consequently, also the child represent a pseudogene) a model of sequence evolution suitable for pseudogenes is used. These models can be varied, but here we use two codon models described below.

In order to model the two modes of sequence evolution, we use two codon substitution matrices proposed by [23], one for the evolution of pseudogenes and other for that of genes. The instantaneous substitution rate matrix from codon *i *to codon *j, q_ij _*is in both cases determined by:

$$q_{ij}=\left\{\begin{array}{ccc} 0, & & \text{if i and j differ at more than one position in a codon triplet}\\ \mu \pi_{j}, & & \text{differ by a synonymous transversion}\\ \mu \kappa \pi_{j}, & & \text{differ by a synonymous transition}\\ \mu \omega \pi_{j}, & & \text{differ by a nonsynonymous transversion}\\ \mu \kappa \omega \pi_{j}, & & \text{differ by a nonsynonymous transition} \end{array}\right)$$

where *π_j _*is the equilibrium frequency of codon j, *μ *is a normalizing factor, *κ *is the transition/transversion ratio, and *ω *is the non-synonymous to synonymous (dN/dS) ratio. Except from *ω*, these parameters are shared between the two modes of sequence evolution. For pseudogenes, *ω *is equal to 1 and transition to stop codons is allowed, whereas for genes transition to stop codon is not allowed.

### The PrIME-PDLRS MCMC framework

PrIME-PDLRS is an MCMC based analysis tool for the above mentioned model. It takes as input a multiple sequence alignment of gene and pseudogene sequences together with a classification of these sequences as genes or pseudogenes. It also requires a dated species-tree *S*. Let us denote a gene-tree by *G*, its edge lengths by *l*, and other parameters of the model by *θ*. The parameter *θ *is compound, containing: the duplication rate; loss rate; pseudogenization rate; edge rate mean and coefficient of variation; and non-synonymous to synonymous rates (dN/dS) and transition/transversion rates for codon substitution model of sequence evolution.

We will use Ψ to denote the set of pseudogenization vertices (degree two) in the gene-tree (no two of these vertices may lie on the same root to leaf path). We use *P*(·) to denote a probability and *p*(·) to denote a probability density.

A state in our Markov chain is a quadruple (*G, l, θ*, Ψ). The leaves in the gene-tree correspond to the given sequences and any sequence classified as a pseudogene must have an ancestor in *G *that belongs to Ψ. When the current state is (*G, l, θ*, Ψ), the acceptance probability of a proposed state $\left(G^{\prime },l^{\prime },\theta^{\prime },\psi^{\prime }\right)$, is determined by the ratio between *p*(*G, l, θ*, Ψ| *D, S*) and $p\left(G^{\prime },l^{\prime },\theta^{\prime },\psi^{\prime }|D,S\right)$, where *D *is the given data and *S *is the species-tree with time. Since each of these densities can be expressed using Bayes equality, e.g.,

$$p\left(G,l,\theta ,\psi |D,S\right)=\frac{P\left(D|G,l,\psi \right)p\left(G,l,\psi |\theta ,S\right)p\left(\theta \right)}{P\left(D|S\right)},$$

the two denominators *P*(*D|S*) in the acceptance probability cancel each other and we obtain

$$\frac{p\left(G,l,\theta ,\psi |D,S\right)}{p\left(G^{\prime },l^{\prime },\theta^{\prime },\psi^{\prime }|D,S\right)}=\frac{P\left(D|G,l,\psi \right)p\left(G,l,\psi |\theta ,S\right)p\left(\theta \right)}{P\left(D|G^{\prime },l^{\prime },\psi^{\prime }\right)p\left(G^{\prime },l^{\prime },\psi^{\prime }|\theta^{\prime },S\right)p\left(\theta^{\prime }\right)}.$$

Here the numerator and denominator have the same structure, so it is sufficient to describe how to compute the former. First, the factor *P*(*D|G, l*, Ψ) can be computed using the dynamic programming (DP) algorithm proposed by Felsenstein [24]. The edges and parts of edges for which the gene or pseudogene mode of sequence evolution should be used is specified by Ψ. The equilibrium frequencies are estimated from the gene and pseudogene sequences, and are shared by both models of sequence evolution. Second, the prior *p*(*θ*) is chosen so that it can be easily computed. Finally, the main technical contribution of [22] is a DP algorithm for computing the likelihood of a gene-tree and its edge lengths given parameters and the species-tree under the DL model. In order to compute *p*(*G, l, θ*, Ψ*|D, S*), we propose a new DP algorithm that integrates the process of pseudogenization and the DL process.

In [22], a DP algorithm for computing the factor *p*(*G, l*|*θ, S*) was described. Let us first define some key concepts. Let *S' *be a discretized species-tree where edges of the species-tree *S *have been augmented with additional discretization vertices such that all the augmented vertices are equidistant within an edge, see figure S1 in additional file 1. The DP makes use of a table, *s*(*x, y, u*), defined as the probability that when a single gene lineage starts to evolve at the vertex $x\in V\left(S^{\prime }\right)$, the tree *G_u _*(the gene-tree rooted at *u *together with the parental edge of *u*) is generated together with the edge lengths specified by *l *and, moreover, the event corresponding to *u *occurs at $y\in V\left(S^{\prime }\right)$. Let *v *and *w *be children of *u *in *G*, and let *x, y*, and z be vertices of *V*(*S'*).

Let *ρ*(*r*) be the probability that an edge of *G *has rate *r*. Also, let *t*(*x, y*) be the time between vertices $x,y\in V\left(S^{\prime }\right)$. Let *σ*(*u*) be the function defined as follows (i) for a leaf u∈L(G), *σ *(*u*) is the species-tree leaf in which the gene that *u *represents can be found and (ii) for any internal vertex *u *of *G, σ *(*u*) is the most recent common ancestor of *L*(*G_u_*) in *S*. We use *p*_11_(*x, y*) to denote the probability of a gene lineage evolving "1-to-1" between two points in the species-tree, i.e., a single gene starting at *x*, for some *k *gives rise to *k *lineages at *y *of which *k *- 1 will go extinct and one gene lineage may or may not go extinct. We use $p_{11}^{\psi }\left(x,y\right)$ to denote the probability of a pseudogene evolving "1-to-1" between two points *x *and *y *in the species-tree, i.e., that a single pseudogene starting at *x*, for some *k *gives rise to *k *pseudogene lineages at *y *of which *k *- 1 will go extinct and one lineage which may or may not go extinct. A vertex u∈V(T) is called a pseudogene if it has an ancestor that belongs to All the vertices representing pseudogenization events Ψ have degree two. How to compute both these "1-to-1" probabilities is described in additional file 1. The following recursions describe how the table *s *can be computed using Dynamic Programming:

1 If u∈L(G) and x = σ(*u*), *s*(*x, x, u*) = 1.

2 If x∈V(S) and x ≠ σ(*u*), *s*(*x, x, u*) = 0.

3 If x∈V(S)\L(S),u∉ψ, and *x *= σ(*u*),

$$s\left(x,x,u\right)=\left(\underset{y\in D_{L}\left(x\right)}{\sum }s\left(x,y,v\right)\right)\left(\underset{y\in D_{R}\left(x\right)}{\sum }s\left(x,y,w\right)\right),$$

where *D*_*L*_(*x*) and *D*_*R*_(*x*) are the descendants of the left and the right child of *x *in *S*', respectively.

4 If $x\in V\left(S^{\prime }\right)\backslash V\left(S\right)$ and u∉ψ,

$$s\left(x,x,u\right)=2\delta \left(\underset{y\in D\left(x\right)\backslash \left\{x\right\}}{\sum }s\left(x,y,v\right)\right)\left(\underset{y\in D\left(x\right)\backslash \left\{x\right\}}{\sum }s\left(x,y,w\right)\right),$$

where *D*(*x*) is the set of descendants of *x*.

5 If x∈V(S), parent of *u *(i.e. *p*(*u*)) is not a pseudogene, and *z *is a child of *x *such that $\sigma \left(L\left(G_{u}\right)\right)\subseteq K\left(S_{z}^{\prime }\right)$ and *z *is an ancestor of *y*, then

$$s\left(x,y,u\right)=p_{11}\left(x,z\right)\varepsilon \left(x,\bar{z}\right)\frac{\rho \left(l\left(p\left(u\right),u\right)/t\left(x,y\right)\right)}{\rho \left(l\left(p\left(u\right),u\right)/t\left(z,y\right)\right)}s\left(z,y,u\right),$$

where $\varepsilon \left(x,\bar{z}\right)$ is the probability that a gene lineage starting at *x *does not reach any leaf $l\in L\left(S_{x}^{\prime }\right)\backslash L\left(S_{z}^{\prime }\right)$. However, if moreover *y *is a child of *x *the above expressions reduce to,

$$s\left(x,y,u\right)=p_{11}\left(x,y\right)\varepsilon \left(x,\bar{y}\right)\rho \left(l\left(p\left(u\right),u\right)/t\left(x,y\right)\right)s\left(y,y,u\right).$$

6 If x∈V(S), *p*(*u*) is a pseudogene, and z is a child of *x *such that $\sigma \left(L\left(G_{u}\right)\right)\subseteq L\left(S_{z}^{\prime }\right)$ and z is an ancestor of *y*, then

$$s\left(x,y,u\right)=p_{11}^{\psi }\left(x,z\right)\varepsilon \left(x,\bar{z}\right)\frac{\rho \left(l\left(p\left(u\right),u\right)/t\left(x,y\right)\right)}{\rho \left(l\left(p\left(u\right),u\right)/t\left(z,y\right)\right)}.$$

However, if moreover *y *is a child of *x *the above expressions reduce to,

$$s\left(x,y,u\right)=p_{11}^{\psi }\left(x,y\right)\varepsilon \left(x,\bar{y}\right)\rho \left(l\left(p\left(u\right),u\right)/t\left(x,y\right)\right)s\left(y,y,u\right).$$

The probability that the gene-tree *G *is generated is the probability that when a single lineage starts at the root of *S*, the single child c of the root of *G *occurs somewhere below the degree one root *ρ *of *S*, and then the process continues and generates *G*. Hence,

$$p\left(G,l|\theta ,\psi ,S\right)=\underset{y\in D\left(\rho \right)}{\sum }s\left(\rho ,y,c\right),$$

where *D*(*ρ*) is the set of descendants of *p*.

#### Sampling d-realizations

In order to map the pseudogenization vertices to the vertices of discretized species-tree *S'*, we use the dynamic programming algorithm proposed in [25]. By suppressing the pseudogenization vertices Ψ of a gene-tree *G *(i.e., removing each degree-two vertex and making its endpoints adjacent), we obtain a gene-tree G*. The sampling algorithm introduced in [25] is used to map the vertices of the gene-tree *V*(G*) to the vertices of the discretized species-tree *V*(*S′*) (see Additional File 1). The time points associated with the vertices of the discretized species-tree, induce an association of time points to the vertices of G*. Once the time points have been associated with the parental vertex and child vertex of a pseudogenization vertex *u *of *G*, a time point can easily be associated with *u*, using the branch lengths of the incident edges.

#### Comparing pseudogenization configurations

We are interested in quantifying the difference between two pseudogenization configurations *G *together with *ψ *and *G′ *together with *ψ^′ ^*of a single gene family. Notice that if we suppress the vertices *ψ *in *G *and *ψ^′ ^*in *G^′ ^*(i.e., remove each such degree-two vertices and make its endpoints become adjacent), respectively, then the same tree G^* ^is obtained. Let *E_ψ _*and *E_ψ′ _*be the set of edges of G^* ^introduced by suppressing *ψ *and *ψ′*, respectively. If the edge *e ∈ E*(G*) was created by suppressing *u*, then *u *is called the origin of *e*.

Notice, for any edge *f *in *E_ψ _*or *E_ψ′_*, all leaves below *f *are pseudogenes. So, if *f ∈ E_ψ_*, then there are either edges of *E_ψ′ _*below *f *on any path from *f *to the leaves below it or there is an edge above *f *that belongs to *E_ψ′_*. In the former case, we call *f *a roof and the edges of *E_ψ′ _*its shade. In the latter case the edge of *E_ψ′ _*is called a roof and *f *belongs to its shade.

The first distance, edge distance, disregards time and is instead defined based on distance in G*. For each pair of edges of G*, there is a unique shortest path containing them; the distance between two such edges is defined to be the number of internal vertices on that path.

**Figure 2:**
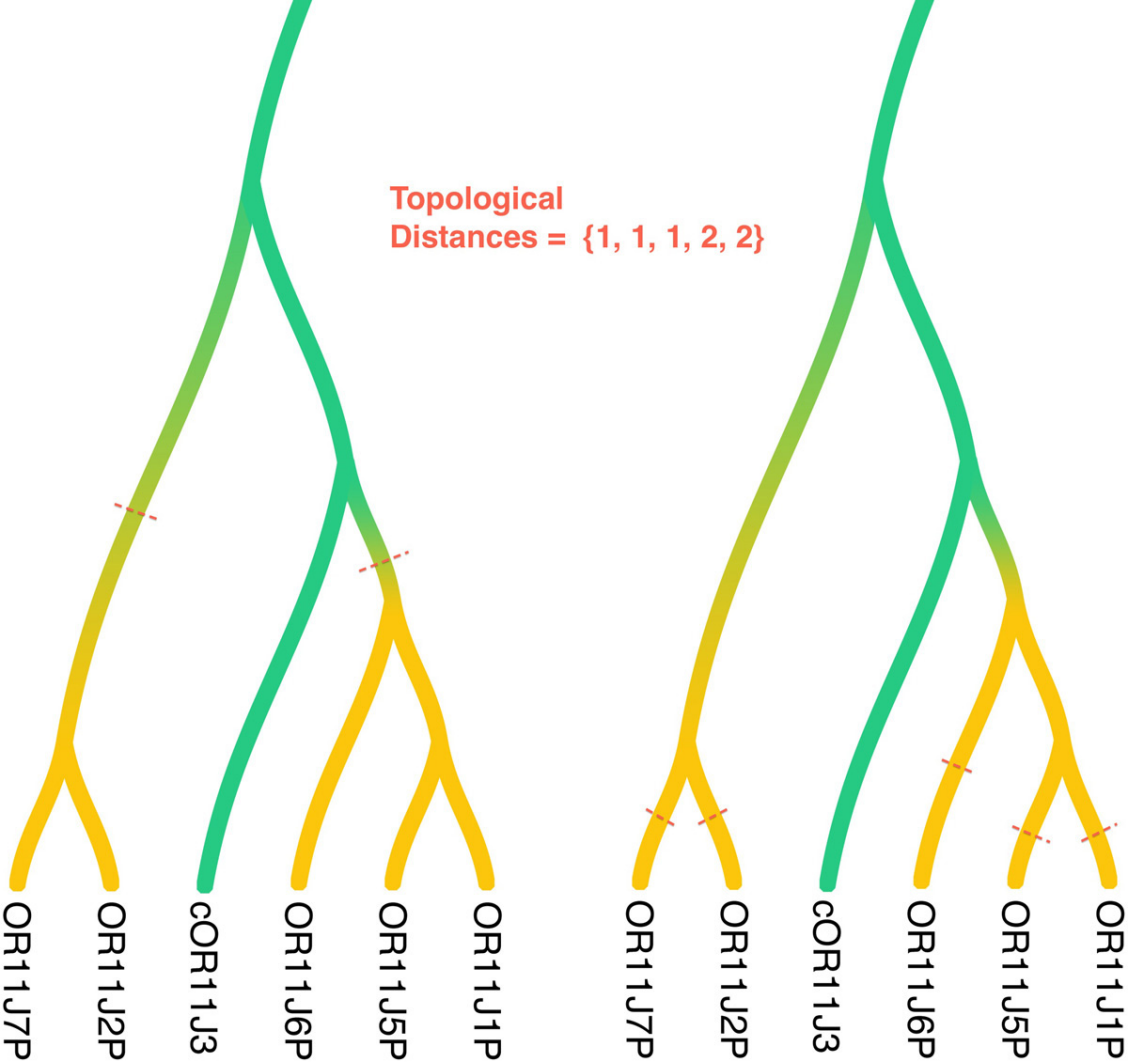
**Topological Distances between two pseudogenization configurations, *D_a _*= ((1 + 1)*/*2 + (1 + 2 + 2)*/*3)*/*2, *D_m _*= *max*(*max*(1, 1)*, max*(1, 2, 2))**.

First, we define two topological distances (Figure 2). The edge distance between two pseudogenization vertices *a_ψ _*and *b_ψ′ _*where *a_ψ_, b_ψ _*are origins of edges *e_a _*and *e_b_*, respectively, such that *e_a_, e_b _∈ E*(*G^∗^*), is defined as the minimum length path between *e_a _*and *e_b _*in *G^∗^*. For each roof edge *f ∈ E_ψ _*or *f ∈ E_ψ′_*, let *d_m_*(*f *) and *d_a_*(*e*) be the maximum edge distance and average edge distance, respectively, between *f *and the edges of its shade. Let the maximum topological distance *D_m _*and average topological distance *D_a _*between *G, ψ *and *G^′^, ψ^′ ^*be the maximum of *d_m_*(*f *) and the average of *d_a_*(*f *), respectively, over all roofs *f ∈ E_ψ _∪ E_ψ′_*. Let the true gene tree and its pseudogenization vertices be (*G, ψ*) and *q *be the posterior probability distribution. Finally, we compute the expected average *E_D_a__* and maximum average *M_D_a__* of the topological distances as:

$$\begin{array}{c} E_{D_{a}}\left(\left(G,\Psi \right),q\right)=\underset{G^{\prime },\Psi^{{}^{\prime }}}{\sum }D_{a}\left(\left(G,\Psi \right),\left(G^{\prime },\Psi^{{}^{\prime }}\right)\right)q\left(G^{\prime },\Psi \right)\\ M_{D_{a}}\left(\left(G,\Psi \right),q\right)=\underset{G^{\prime },\Psi^{{}^{\prime }}}{\text{max}}D_{a}\left(\left(G,\Psi \right),\left(G^{\prime },\Psi^{{}^{\prime }}\right)\right)q\left(G^{\prime },\Psi \right) \end{array}$$

We also define the expected maximum *E_D_m__* and maximum maximum *M_D_m__* of the topological distances as:

$$\begin{array}{c} E_{D_{m}}\left(\left(G,\Psi \right),q\right)=\underset{G^{\prime },\Psi^{{}^{\prime }}}{\sum }D_{m}\left(\left(G,\Psi \right),\left(G^{\prime },\Psi^{{}^{\prime }}\right)\right)q\left(G^{\prime },\Psi \right)\\ M_{D_{m}}\left(\left(G,\Psi \right),q\right)=\underset{G^{\prime },\Psi^{{}^{\prime }}}{\text{max}}D_{m}\left(\left(G,\Psi \right),\left(G^{\prime },\Psi^{{}^{\prime }}\right)\right)q\left(G^{\prime },\Psi \right) \end{array}$$

Second, we define the temporal distances. These are obtained analogously to the topological, but instead of using the edges distances between roofs and their shades, we use the temporal distances between the time associated with the origin of a roof and the time associated with the origins of its shade.

Topological distance measures the distance of a true pseudogenization vertex from the inferred one along the gene tree topology, whereas the temporal distance measures the distance between the times (along the species tree) associated with the true pseudogenization vertex and the inferred one.

### Synthetic and Biological Analysis

We tested our method PrIME-PDLRS on synthetic data and applied it to biological data. We first describe the tests on synthetic data. Random gene-trees with edge lengths and pseudogenization vertices were generated using a modified version of PrIME-Gene-Tree generator [26] with pseudogenization rate of 0.5, and biologically realistic duplication-loss rates observed by analyzing gene families of OPTIC dataset [27]. Gene sequences were generated according to the PDLRS model. Gene sequences were evolved using codon substitution matrices as proposed by Bielawski et al. [23]. A neutral codon substitution matrix was used for the evolution of pseudogenes where the rate ratio of non-synonymous to synonymous substitutions (dN/dS) was set to 1.0. In the neutral codon substitution model, any codon could be substituted with a stop codon, while this was not possible under the substitution model used in the case of gene evolution. Twenty five different combinations of dN/dS rate ratios and transition/transversion rate ratios were used to generate gene sequences across twenty five gene families, using uniform codon equilibrium frequencies. In order to simulate a biologically realistic scenario, we used the species-tree (obtained as in [25]) for the nine vertebrate species of OPTIC [27] dataset, which was downloaded from http://genserv.anat.ox.ac.uk/downloads/clades/ The inferred pseudogenization vertices were then compared with the true pseudogenization vertices using two kinds of distance metrics, i.e. topological distance (gene-tree), and temporal distance (species-tree).

The biological datasets consisted of sub-families from the two largest gene families of vertebrates, i.e. olfactory receptors and zinc fingers. Olfactory receptors have been reported to be the largest gene family in the vertebrates [28]. In species such as cow, platypus, and primates, a high rate of pseudogenization has been observed, while opossum, dogs, mouse and rats have relatively low rate of pseudogenization [28]. Seven sub-gene families preferably having at least one pseudogene per species were downloaded from http://bioportal.weizmann.ac.il/HORDE/ for the species of human (Homo sapiens), dog (Canis lupus familiaris), opossum (Didelphis virginiana), and platypus (Ornithorhynchus anatinus). Two zinc finger sub-gene families were also studied across the species of human (Homo sapiens), chimpanzee (Pan troglodytes), orangutan (Pongo abelii), and rhesus macaque (Macaca mulatta). For this purpose, we chose two sub-families from the the high confidence orthologous genes (which are supported by OrthoMCL [29], reciprocal best BLAST hits, and synteny). The corresponding parent/paralogous genes were searched using PSI-BLAST [30] and extracted from http://ensembl.org. The high confidence orthologous genes were downloaded from 'The KZNF catalog' (http://znf.igb.illinois.edu) [31,32]. As the pseudogenes in the zinc finger gene family have mostly evolved as a result of fragmented duplications [31], it is challenging to correctly align the pseudogenes and corresponding genes, clearly a necessary condition for reconstructing the gene-tree. Alignments of the nine sub-gene families were manually curated after aligning them with MACSE [33], allowing stop codons and introducing penalties for creation of a gap (-7), extending a gap (-1), and introducing frameshift (-14). The dated species-trees for both the biological datasets were downloaded from http://timetree.org[34]. The sub gene-families were then analyzed using the same pipeline as used for synthetic analysis. Potential gene-trees were reconstructed using PrIME-DLRS, which were then analyzed by PrIME-PDLRS using fixed gene-tree option. The PrIME-DLRS gene-tree having best PrIME-PDLRS state with the highest posterior probability was selected as the most likely gene-tree. The posterior over pseudogenization events of the most likely gene-trees were then analyzed using the detailed-realizations generated during the Markov chain traversal.

### MCMC Analysis

Bayesian analysis was performed for the gene families using MCMC based analysis tool, PrIME-PDLRS. The MCMC chain was setup to integrate over all the parameters, i.e. gene-tree, edge lengths, pseudogenization vertices on gene-tree, birth-death and pseudogenization rates, and mean and variance of edge substitution rates. We sampled different parameters throughout the MCMC process including birth-death rates, pseudogenization rate, gene-tree, pseudogenization vertices, dN/dS rate ratio, and transition/transversion rate ratio. One or more parameters were perturbed at each iteration. The perturbation of the gene-tree was done using standard gene-tree perturbation methods such as subtree pruning and regrafting, nearest neighbor interchange and re-rooting. After a perturbation, the validity of the resulting gene-tree was certified, i.e., no pseudogene lineage lead to a gene lineage. A valid perturbed gene-tree is proposed, every time a gene-tree is proposed. Neighbor Joining method [35] is used to construct the initial tree at the start of MCMC chain. The proposal distribution proposes moves of pseudogenization vertices, across the lineages of a gene-tree, in a manner such that the probability of proposing an upward move of a pseudogenization vertex is equal to the probability of proposing a downwards move. The dN/dS rate ratios are sampled from a truncated normal distribution in [0, 10], while the transition/transversion rate ratios are sampled from a truncated normal distribution in [0-100]. The birth-death and pseudogenization rates are sampled from a truncated normal distribution in [0, *inf *]. Truncated normal proposals were used for the perturbation of the parameters of the rate model and edge lengths around the current value, with tuning parameters handcrafted with respect to acceptance ratios. Substitution rate parameters were perturbed by either perturbing the distribution mean or the coefficient of variation. In order to find if the MCMC chains have converged, we used VMCMC [36] as a diagnostic tool. From the initial runs, it was observed that it was safe to use a burn-in period of 2,500,000. For the rest of the runs, we used 5,000,000 iterations, burn-in period of 2,500,000 and thinning of 500. We used PrIME-DLRS as a first step to reconstruct the potential gene-trees. Each potential gene-tree was analyzed using PrIME-PDLRS with a fixed gene-tree option.

## Results

PrIME-PDLRS was first tested on a synthetic dataset, and then used for analyzing biological gene-families. First we discuss the results for the synthetic data, and then we will discuss the biological results. In the case of synthetic data, distances between the true pseudogenization vertices were measured using two different kinds of distance metrics as described above. The total time from root to leaf on the species-tree is scaled to 1.0, which corresponds to 400 million years on evolutionary time scale (synthetic data).

In nineteen of the synthetic gene families PrIME-PDLRS was able to identify the true gene-tree and the average topological distance was less than 1.0 on average across the posterior. In fifteen gene families the maximum topological distance was also less than 1.0 on average across the posterior. PrIME-PDLRS also performed well in terms of the temporal distance. In fifteen of the gene families, the average temporal distances between the true and inferred configuration was less than or equal to 0.16 on average across the posterior (see table 1). The maximum temporal distance was found to be less than or equal to 0.38 in 16 synthetic families. The values of *ω *and *κ *seems to have no significant effect on the topological and temporal distances between the true and inferred pseudogenization vertices. The method could not infer the true gene-tree for two of the synthetic gene families.

**Table 1:** Topological (avg: *d_a_*, max: *d_m_*) & Temporal Distances (avg: *t_a_*, max: *t*_*m*_) of the inferred pseudogenization vertices from the true (synthetic analysis)

Family	mean(*d_a_*)	mean(*t_a_*)	max(*d_m_*)	mean(*d_m_*)	max(*t_m_*)	mean(*t_m_*)	dN/dS	Kappa	Depth
1	0	0.03	0	0	0.13	0.03	0.3	0.2	1

2	1.12	0.34	3	3	0.86	0.68	0.3	0.8	6

3	0	0.03	0	0	0.14	0.04	0.3	1.2	2

4	0	0.22	0	0	0.63	0.5	0.3	1.6	1

5	0.56	0.12	3	3	0.33	0.24	0.3	2	4

6	1.3	0.13	3	3	0.4	0.23	0.6	0.2	4

7	0	0.03	0	0	0.19	0.03	0.6	0.8	1

8	0	0.08	0	0	0.26	0.15	0.6	1.2	3

9	0.1	0.04	1	0.1	0.12	0.04	0.6	1.6	2

10	0	0.16	0	0	0.31	0.25	0.6	2	7

11	0	0.14	0	0	0.28	0.2	0.8	0.2	2

12	0	0.05	0	0	0.23	0.08	0.8	0.8	3

13	0.35	0.32	1	0.69	0.54	0.38	0.8	1.2	6

14	0	0.03	0	0	7.0	0.03	0.8	1.6	3

17	0.66	0.41	1	1	0.76	0.73	1.2	0.8	3

18	0.88	0.39	1	0.88	0.67	0.43	1.2	1.2	5

19	0	0.15	0	0	0.21	0.15	1.2	1.6	1

20	0	0.11	0	0	0.27	0.22	1.2	2	3

21	0.33	0.25	1	1	0.65	0.53	1.8	0.2	3

22	1.4	0.42	4	4	0.81	0.78	1.8	0.8	5

23	1.03	0.11	3	1.23	0.3	0.14	1.8	1.2	4

24	0.67	0.27	1	1	0.67	0.43	1.8	1.6	3

25	0.04	0.13	3	0.04	0.45	0.13	1.8	2	6

**Figure 3:**
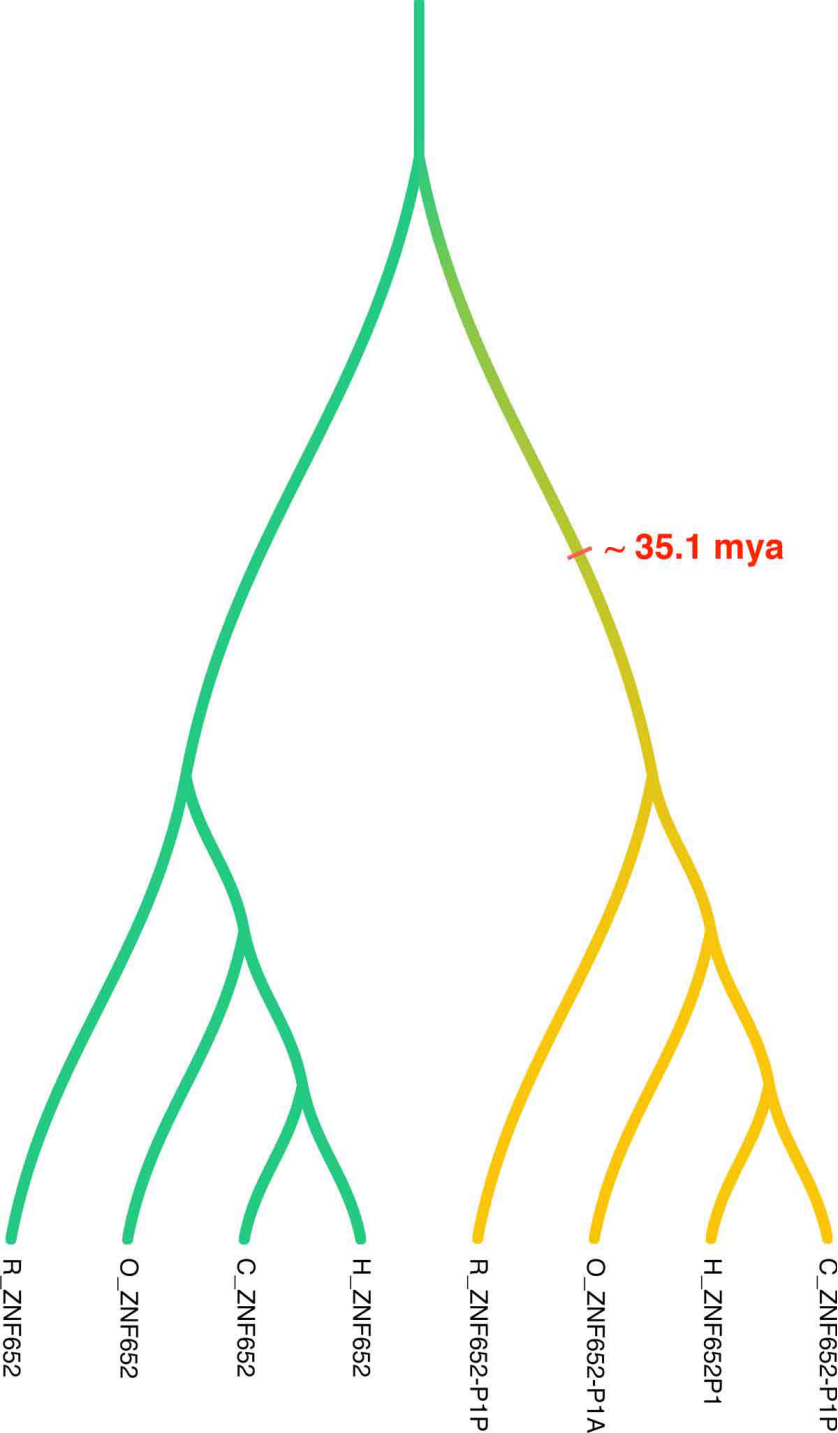
**ZNF652 (Zinc Fingers sub-gene family)**.

We took two zinc finger sub-families, ZNF652 and SNAI1, across four species of Homo sapiens (human), Pan troglodytes (chimpanzee), Pongo abelii (orangutan) and Macaca mulatta (rhesus macaque), and analyzed them using PrIME-PDLRS. The evolutionary history of ZNF652 (ZNF subfamily) is illustrated in Figure 3. This gene family appears to have a signal in favor of early pseudogenization of the sub-gene-tree formed by pseudogenes; the pseudogenization vertices were mostly mapped to the ancestral gene lineage of all the four pseudogenes (across four species). In the other gene family, SNAI1, in most cases the pseudogenization events were mapped to the ancestral lineage of the two pseudogenes present only in human and chimpanzee (Figure S2). So both these zinc finger families support early pseudogenization events. Assuming these pseudogenes of ZNF652 evolved as non-functional pseudogenes before the split of rhesus and human, their conservation is surprisingly high. Using the neutral rate of evolution (estimated by analyzing multiple sequence alignment of nonfunctional regions of the four species), we computed the P-value of the conservation of ZNF652 subfamily (similar to [37]). Although the P-value of the conservation was low, it was not significant. We also attempted to investigate several other zinc finger sub-families, but since most of the ZNF pseudogenes arose from the neighboring loci through partial-gene duplication [31], their alignment as well as gene-tree reconstruction are highly challenging tasks.

We also analyzed some sub-families from the olfactory receptors gene family. The species considered in the case of olfactory receptors were human, dog, opossum, and platypus. The species considered in this case are much more ancient than the primates considered for zinc finger gene family. In this case, we estimated some ancient pseudogenization events as well as relatively recent ones. Figure S3 illustrates one of the gene families analyzed by PrIME-PDLRS. In the gene family OR10B (Figure S3), approximately 90% of the time the pseudogenization vertices were sampled on gene lineages incident to leaves (OR10B1P (human), cOR10B1P (dog)). The pseudogenization vertices were 65-70 million years old according to the times sampled for the pseudogenization vertices. OR2W is another sub-gene family (Figure S4) where some of the pseudogenization vertices were estimated to be recent, while others were quite old. An example of the relatively recent pseudogenization events is the sub-gene-tree consisting of platypus genes oa-OR2W21P, oa-OR2W22P, and oa-OR2W23P that was estimated to have independently pseudogenized after the duplications of the ancestral gene lineage. Pseudogene OR2W2P was estimated to have pseudogenized around 104 mya even before the time associated with human-dog speciation split. The most ancient pseudogene estimated in the analysis is the opossum gene Modo-OR2W2P, which is estimated to have pseudogenized even before the time associated with human-platypus split around 180 million years ago (mya). Both recent and ancient pseudogenization events were estimated for OR2B (Figure S5). The sub-gene-tree consisting of platypus genes oa-OR2B14P, oa-OR2B15P, oa-OR2B16P, oa-OR2B17P and oa-OR2B18P was also estimated to have independently pseudogenized after being duplicated as genes. The most ancient pseudogene in this sub-family was human gene OR2B26 that is estimated to have pseudogenized around 118 million years ago (before the time associated with human-dog speciation split). In human sub-gene family OR11J (Figure S6), there are two sub-gene-trees that were estimated to have pseudogenization events higher in the sub-gene-tree (40-60 mya). One such sub-gene-tree consists of human genes OR11J1P, OR11J5P and OR11J6P which has pseudogenized around 60 mya after the time associated with human-dog speciation split and then duplicated as pseudogenes. Similarly, another lineage leading to the sub-gene-tree consisting of human genes OR11J2P and OR11J7P was estimated to have pseudogenized around 42 mya, which then duplicated as pseudogenes. Two other pseudogenes cOR11J4P and oa-OR11J11P were estimated to have first duplicated and then pseudogenized around 125-130 mya before the human-dog speciation split. A dog specific sub-gene-tree where the pseudogenization vertices were estimated on the ancestral edge of the sub-gene-tree (cOR1D9P and cOR1D10P) around 38 mya was also analyzed for the sub-gene family OR1D (Figure S7). This sub-family also have interesting dog and platypus pseudogenes that are estimated to have pseudogenized around 80 mya and 135 mya, respectively. In OR9I, opossum also had two sub-gene-trees, where most of the pseudogenization vertices were estimated on the ancestral lineage of the two pseudogenes leaves (Figure S8). The time estimates show that they have pseudogenized before the human-dog speciation split around 75 mya and 103 mya. Another interesting sub-gene-tree consisting of OR9I3P (human) and cOR9I4P (dog) is estimated to have pseudogenized independently after duplicating as a gene lineage. The time estimates shows that the lineages pseudogenized around 75-80 mya. Relatively recent pseudogenization events were estimated in the sub-gene family OR4K (Figure S9). The only ancient pseudogenes in this sub-family are human genes OR4K16P, OR4K4P, and opossum gene Modo-OR4K15P that are estimated to have pseudogenized around 80 mya.

## Discussion

Olfactory receptors are known for their high rate of pseudogenization in primates [28]. The reasons for the high rate of pseudogenization include the evolution of sophisticated abilities to sense their environment such as trichromatic vision in primates. It has been suggested that the expansion of the olfactory receptor gene family has occurred during the process of terrestrial adaptation in the tetrapod lineage and continued until the mammalian radiation [28,38]. Aquatic or semiaquatic animals are also known for high rate of pseudogenization, because of the evolution of alternative means to sense the environment. Platypus is a species that can sense the environment and find the prey through a sophisticated combination of electroreception and mechanoreception [28]. Zinc fingers is another gene family that has a high rate of pseudogenization. It is the second largest gene family in the human genome. The Zinc Finger motif is the most common DNA binding motif found in eukaryotes. Zinc fingers are thought to have expanded and diversified through segmental duplications [39]. In this study, we explore some sub-gene families of the two gene families in the human genome. We proposed here a probabilistic method to estimate the age of pseudogenes and analyzed nine sub-families of the two mentioned gene families.

We analyzed seven olfactory receptor gene families across four species (human, dog, opossum and platypus). We expected to find some ancient pseudogenes, as the expansion of OR genes was reported to have occurred in the tetrapod lineage. Although the majority of pseudogenes were not very old, we were able to find some ancient pseudogenes. Pseudogenes that are conserved to some degree and are not lost at such large evolutionary distances are interesting and could be suggestive of functionality. The oldest pseudogene estimated in our study was in the gene family OR2W, where pseudogenization of the gene Modo-OR2W2P took place before the human-platypus speciation split (approximately 182 mya). This pseudogene lineage is in our study, however, species lineage specific, which may be less trustworthy than pseudogenizations occurring above speciations in the species-tree used in the analysis.

We also found seven pseudogenes that are estimated to have pseudogenized before the time associated with human-dog speciation split. Opossum had two such pseudogenes in OR9I, platypus had two pseudogenes in OR1D and OR11J, human had two pseudogenes in OR2W and OR2B, and dog had one such pseudogene in OR11J. Around 33 pseudogenes across all the selected OR sub-gene families are estimated to have pseudogenized more than 30 mya. Analysis of these seven sub-gene families of olfactory receptors makes OR gene family an interesting candidate for future investigations in pseudogenes.

We also analyzed two sub-gene families of zinc fingers. We were again interested in finding pseudogenes that have evolved as pseudogenes over long period of time on an evolutionary scale. The ZNF652 and SNAI1 sub gene-families were considered in this study. The sub gene-family SNAI1 had two pseudogenes that were estimated to have pseudogenized around 12.6 mya before the human-chimpanzee speciation split. Four non-lineage specific pseudogenes belonging to ZNF652 sub-gene family were estimated to have pseudogenized before the speciation split of human and rhesus. This makes these genes interesting, as they have been conserved across the four species for around 35.1 million years. We analyzed the gene family for conservation (using an analysis similar to that in [37]). Although the P-value was low, it was not significant. We also searched the pseudogenes studied in ncRNA database [40] but found no hits.

Probabilistic methods have been used widely in the phylogenetic studies largely due to their desirable mathematical properties. However, with the increasing sizes of gene families, MCMC convergence over the parameters space can be slow. We believe that our method, being the first probabilistic approach to estimate the age of a pseudogene based on data for extended gene families, provides new opportunities in identification of potentially functional pseudogenes. In future, the availability of pseudogene families across the tree of life will enable us to analyze the evolution of pseudogenes in further detail. We hope that with better analysis of pseudogenes it will be possible to differentiate functionally active pseudogenes from functionally non-active pseudogenes.

## Competing interests

The authors declare that they have no competing interests.

## Supplementary Material

Additional File 1 **Supplementary Information**. Click here for file

## Declarations

The publication charges of this article were supported by the Swedish Research Council (Grant 2013-4993).

This article has been published as part of *BMC Genomics *Volume 16 Supplement 10, 2015: Proceedings of the 13th Annual Research in Computational Molecular Biology (RECOMB) Satellite Workshop on Comparative Genomics: Genomics. The full contents of the supplement are available online at http://www.biomedcentral.com/bmcgenomics/supplements/16/S10.
