# Supplementary material for: Gene-pseudogene evolution: a probabilistic approach
Source: BMC Genomics. 2015 Oct 2;16(Suppl 10):S12. doi: 10.1186/1471-2164-16-S10-S12 (PMC4602177; doi:10.1186/1471-2164-16-S10-S12)
Supplement: Additional File 1 — Supplementary Information. [file 1471-2164-16-S10-S12-S1.pdf]

Supplementary Information

July 31, 2015

Computing probabilities for lineages of pseudogenes

Below we derive differential equations for probabilities that are essential when computing the generation probabilities of a gene tree with lengths according to the PDLRS model. Of course, this probability depends on the birth, death and pseudogenization rates. The probability of extinction of a gene lineage is discussed in the first section, whereas, the "1-to-1" probability of a single gene lineage between two points in time is discussed in the following section.

Extinction probabilities

In the full model, there is a parameter for the loss rate for genes and another parameter for the loss rate of pseudogenes. Below, we use one parameter for both these rates, which simplifies notation and derivations, but it is also desirable when analyzing relatively small instances in order to avoid over-parametrisation. In this section, we will derive a differential equation for the probability of extinction.

Also here G is the gene tree, it is evolving inside a species tree S, the duplication rate is δ, the loss rate is μ, and the pseudogenization rate is ψ. For an infinitesimal interval $[t, t+\Delta]$ (where t is closer to the leaves of S than what $t+\Delta$ is) and an edge e with a time interval containing $[t, t+\Delta]$ there are four possibilities for a lineage entering this interval on this particular edge:

1. a duplication occurs in the time interval $[t, t+\Delta]$, and both of the resulting lineages subsequently go extinct (i.e., at time points $t', t'' \leq t$),
2. the lineage is a gene lineage, a pseudogenization event occurs in the time interval, and the pseudogene subsequently go extinct
3. a loss occurs in the time interval, or
4. no event takes place in the interval.

The extinction probability of a gene lineage evolving in e ∈ E(S) is thus given by:

$$\begin{aligned} &Q_{ex}(e, t+\Delta) \\ &\quad = \delta\Delta(Q_{ex}(e,t))^2 + \Delta\psi Q_{ex}(e,t) + \Delta\mu + (1 - \Delta\psi - \Delta\delta - \Delta\mu)Q_{ex}(e,t) \\ &\quad = \delta\Delta(Q_{ex}(e,t))^2 + \Delta\mu + (1 - \Delta\delta - \Delta\mu)Q_{ex}(e,t) \end{aligned}$$

Notice that, as intuitively clear, the pseudogenization events is inconsequential when genes and pseudogenes have the same loss rate. Rearranging the above equation, and taking the differential gives us the following equation:

$$\frac{d}{dt}Q_{ex}(e,t) = \delta(Q_{ex}(e,t))^2 + \mu - (\delta+\mu)Q_{ex}(e,t)$$

For $e = (u, v) \in E(S)$ where the child v is associated with time t_v, the initial values for the system of equations for Q_{ex} are given by:

$$Q_{ex}(e, t_v) = \begin{cases} 0 & \text{if } v \text{ is a leaf,} \\ Q_{ex}(f, t_v) & \text{if } v \text{ has only one outgoing edge } f, \\ Q_{ex}(f, t_v)Q_{ex}(f', t_v) & \text{if } v \text{ has two outgoing edges } f \text{ and } f'. \end{cases}$$

The extinction probability for a pseudogene lineage is computed by the standard birth-death process similar to [1].

"1-to-1" evolution

In this section, we will derive a differential equation for the probability of a gene lineage evolving "1-to-1" between two points in the species tree not separated by a speciation event (that is, a single gene lineage starts at the first point and has a single descendant at the second point which also is a gene which may not go extinct, while all other descendant will go extinct). We will also comment on how the analogous probability for pseudogenes can be obtained, i.e., the for the probability of a pseudogene lineage evolving "1-to-1" between two points in the species tree not separated by a speciation event (that is a single pseudogene lineage starts at the first point and has a single descendant at the second point, in this case the lineage can only be a pseudogene, which may not go extinct, while all other descendant will go extinct).

We start by considering (1). For an infinitesimal interval and an edge e with a time interval containing $[t, t + \Delta]$ there are the following possibilities for a gene lineage evolving "1-to-1" in this interval on this particular edge:

1. a duplication occurs in the time interval and one of the child lineage subsequently goes extinct
2. no duplication, no pseudogenization, and no loss occurs in the interval, and the lineage continues to evolve "1-to-1" after time interval

Hence, the "1-to-1" probability Q_{11} of a gene lineage between time t and reaches time t' satisfies:

$$Q_{11}(e, t + \Delta, t') = 2\delta\Delta Q_{11}(e, t, t')Q_{ex}(e, t) + (1 - \Delta\delta - \Delta\psi - \Delta\mu)Q_{11}(e, t, t').$$

Rearranging the equation and taking differential give us the following equation:

$$\frac{d}{dt}Q_{11}(e, t, t') = 2\delta Q_{11}(e, t, t')Q_{ex}(e, t) - (\delta + \psi + \mu)Q_{11}(e, t, t')$$

This enables us to compute probability of any gene lineage evolving between two points in time that are not separated by a speciation event.

The probability of "1-to-1" gene evolution between between two vertices x and y, $p_{11}(x, y)$, of a discretized species tree S', where x is the parent of y, is given by $Q_{11}(xy, t_ux, t_y)$, where t_x and t_y are the time points associated with u and v, respectively.

We now consider (2). This case can be handled analogous to how the corresponding probability is handled in [1], that is, as follows. Define $Q_{11}(e, t, t')$ as the probability that a single gene, starting at t on e, has $k + 1$ descendants in y, for some k, of which one may or may not have descendants in the leaves of S while the remaining k go extinct before reaching the leaves of S, i.e,

$$Q_{11}(e, t, t') = \sum_{k=0}^{\infty} P(k \text{ descendant after time } t' - t)(k + 1)Q_{ex}(e, t')^k,$$

where $P(k$ descendant after time $t' - t)$ can be computed using closed forms from [2].

Also in this case, the probability of "1-to-1" gene evolution between between two vertices x and y, $p_{11}^{\Psi}(x,y)$, of a discretized species tree S', where x is the parent of y, is given by $Q_{11}(xy, t_u, t_v)$, where t_x and t_y are the time points associated with x and y, respectively.

The analogous '1-to-1" probability for pseudogenes can be obtained using the function $s(x, y, u)$ introduced in [1] where the pseudogene lineage evolves from a discretization vertex $x \in V(S')$ in the species tree and reaches another discretization vertex $y \in V(S')$ such that all the duplicated pseudogene lineages goes extinct except the pseudogene lineage u.

Sampling d-realization with suppressed pseudogenization events

This algorithm was introduced in [3]. It makes use of a table, $s(x, y, u)$, defined as the probability that when a single gene lineage starts to evolve at the vertex $x \in V(S')$, the tree G_u is generated together with the edge lengths l and, moreover, the event corresponding to u occurs at $y \in V(S')$. Let v and w be children of u in G, and let x, y, z be vertices of $V(S')$. Let $\rho(r)$ be the probability that an edge of G has rate r. Also, let $t(x, y)$ be the time between vertices $x, y \in V(S')$. The following recursions describe how the table s can be computed:

1. If $u \in L(G)$ and $x = \sigma(u)$, $s(x, x, u) = 1$.
2. If $x \in V(S)$ and $x \neq \sigma(u)$, $s(x, x, u) = 0$.
3. If $x \in V(S)\backslash L(S)$ and $x = \sigma(u)$,
$$s(x, x, u) = \Big(\sum_{y \in D_L(x)} s(x, y, v)\Big)\Big(\sum_{y \in D_R(x)} s(x, y, w)\Big),$$
where $D_L(x)$ and $D_R(x)$ are the descendants of left and right child of x in S', respectively.
4. If $x \in V(S)$ and z is a child of x such that $\sigma(L(G_u)) \subseteq L(S'_z)$ and z is an ancestor of y,
$$s(x, y, u) = p_{11}(x, z)\varepsilon(x, \overline{z})\frac{\rho(l(\hat{p}(u), u)/t(x, y))}{\rho(l(\hat{p}(u), u)/t(z, y))}s(z, y, u),$$
where $\varepsilon(x, \overline{z})$ is the probability that a gene lineage starting at x does not reach any leaf $l \in L(S'_x)\backslash L(S'_z)$ and $\hat{p}(u)$ is the parent of u. However, if $y = z$, the expression reduces to the following,
$$s(x, y, u) = p_{11}(x, y)\varepsilon(x, \overline{y})\rho(l(\hat{p}(u), u)/t(x, y))s(y, y, u).$$
5. If $x \in V(S')\backslash V(S)$,
$$s(x, x, u) = 2\delta\Big(\sum_{y \in D(x)\backslash\{x\}} s(x, y, v)\Big)\Big(\sum_{y \in D(x)\backslash\{x\}} s(x, y, w)\Big),$$
where $D(x)$ is the set of descendants of x.
6. If $x \in V(S')\backslash V(S)$ and z is the child of x in the discretized species tree S',
$$s(x, y, u) = p_{11}(x, z)\frac{\rho(l(\hat{p}(u), u)/t(x, y))}{\rho(l(\hat{p}(u), u)/t(z, y))}s(z, y, u).$$
However, if $y = z$, the expression reduces to the following,
$$s(x, y, u) = p_{11}(x, y)\rho(l(\hat{p}(u), u)/t(x, y))s(y, y, u).$$

In any reconciliation or d-realization, the planted root of G is mapped to the planted root of S. The probability that the gene tree G is generated is the probability that when a single lineage starts at the root of S, the root of G occurs somewhere below the planted root of S and then the process continues and generates G. Hence,

$$p(G, l|\theta, S) = \sum_{y \in D(p)} s(p, y, r),$$

where p is the planted root of S, $D(p)$ its descendants, and r is the root of G. Consequently, the probability that r is mapped to $y \in V(S')$ by a d-realization sampled from all d-realizations according to the posterior probability distribution under observed G and l, is

$$\frac{s(p, y, r)}{p(G, l|\theta, S)} = \frac{s(p, y, r)}{\sum_{z \in D(p)} s(p, z, r)}.$$

Similarly, if we know that a d-realization maps a vertex $u \in V(G)$ to a vertex $x \in V(S')$, then the probability that a child v of u is mapped to y by a realization sampled from all such d-realizations, according to the posterior probability distribution under observed G and l, is

$$\frac{s(x, y, u)}{\sum_{z \in D(x)} s(x, z, u)}.$$

This clearly provides an algorithm for sampling d-realizations according the posterior probability distribution under observed G and l.

References

[1] Åkerborg Ö, Sennblad B, Arvestad L, Lagergren J: **Simultaneous Bayesian gene tree reconstruction and reconciliation analysis**. *Proceedings of the National Academy of Sciences* 2009, **106**(14):5714–5719.

[2] Kendall DG: **On the generalized" birth-and-death" process**. *The annals of mathematical statistics* 1948, :1–15.

[3] Mahmudi O, Sjöstrand J, Sennblad B, Lagergren J: **Genome-wide probabilistic reconciliation analysis across vertebrates**. *BMC bioinformatics* 2013, **14**(Suppl 15):S10.

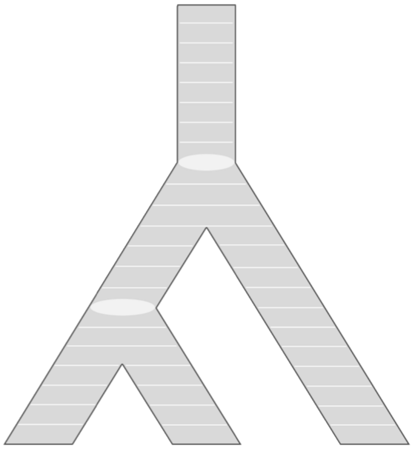

Figure S1: A discretized species tree

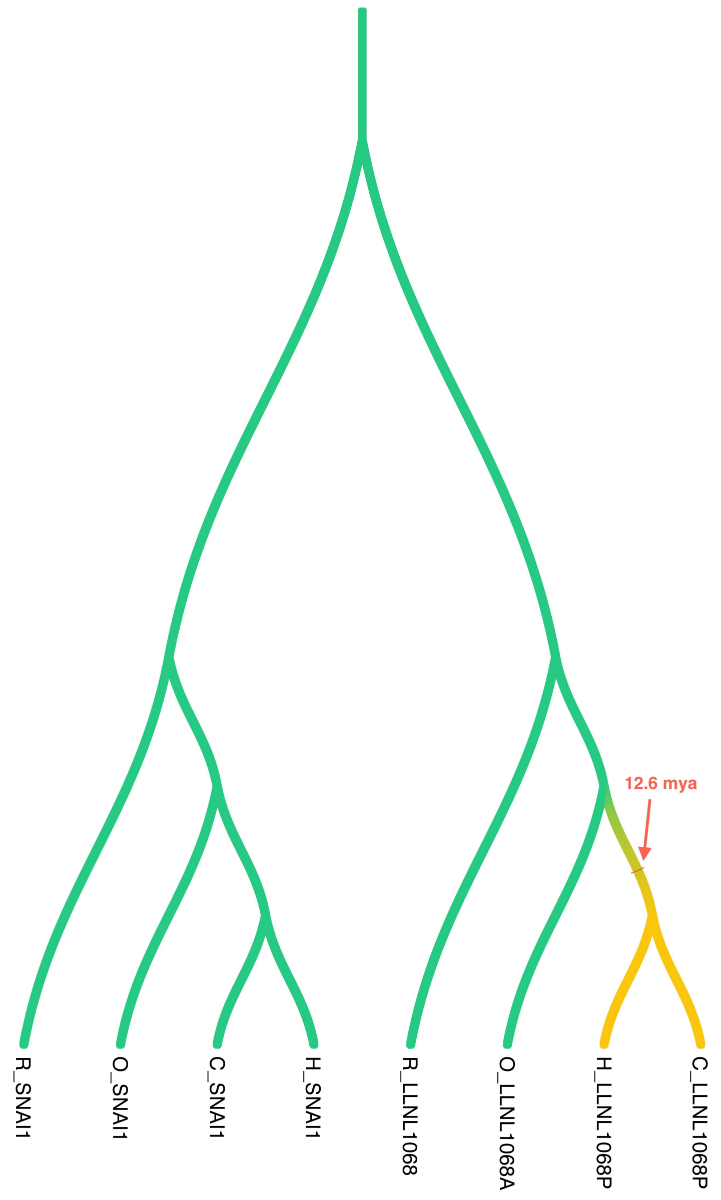

Figure S2: SNAI1 (Zinc Fingers)

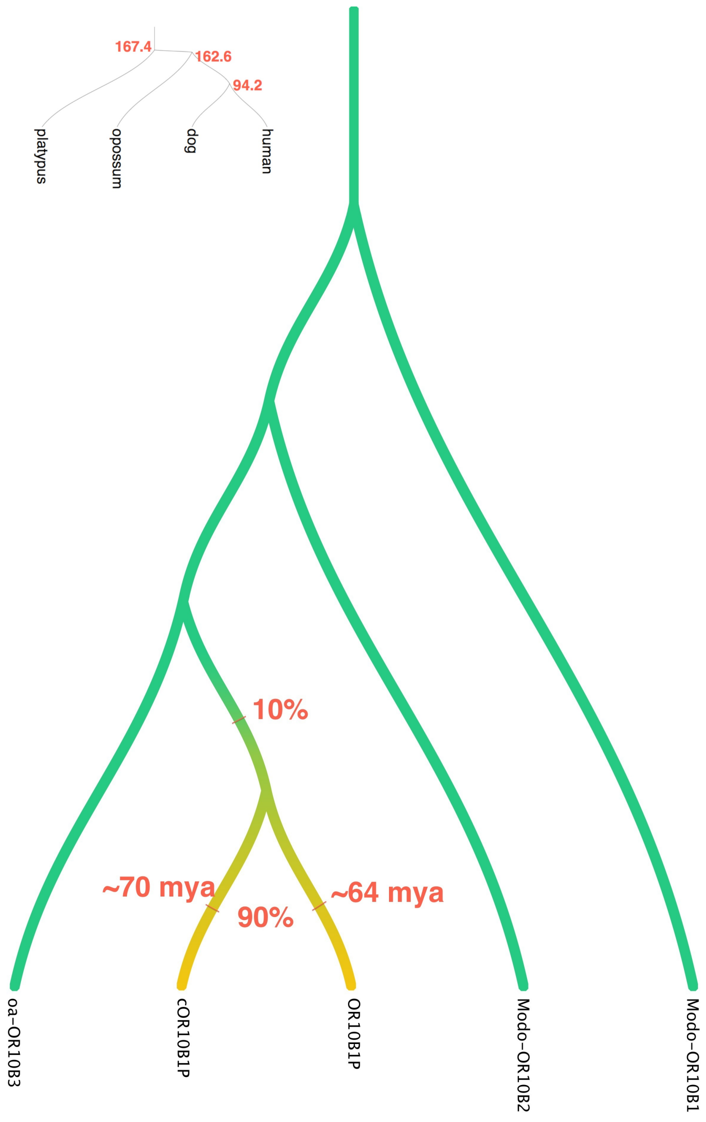

Figure S3: OR10B (Olfactory Receptors)

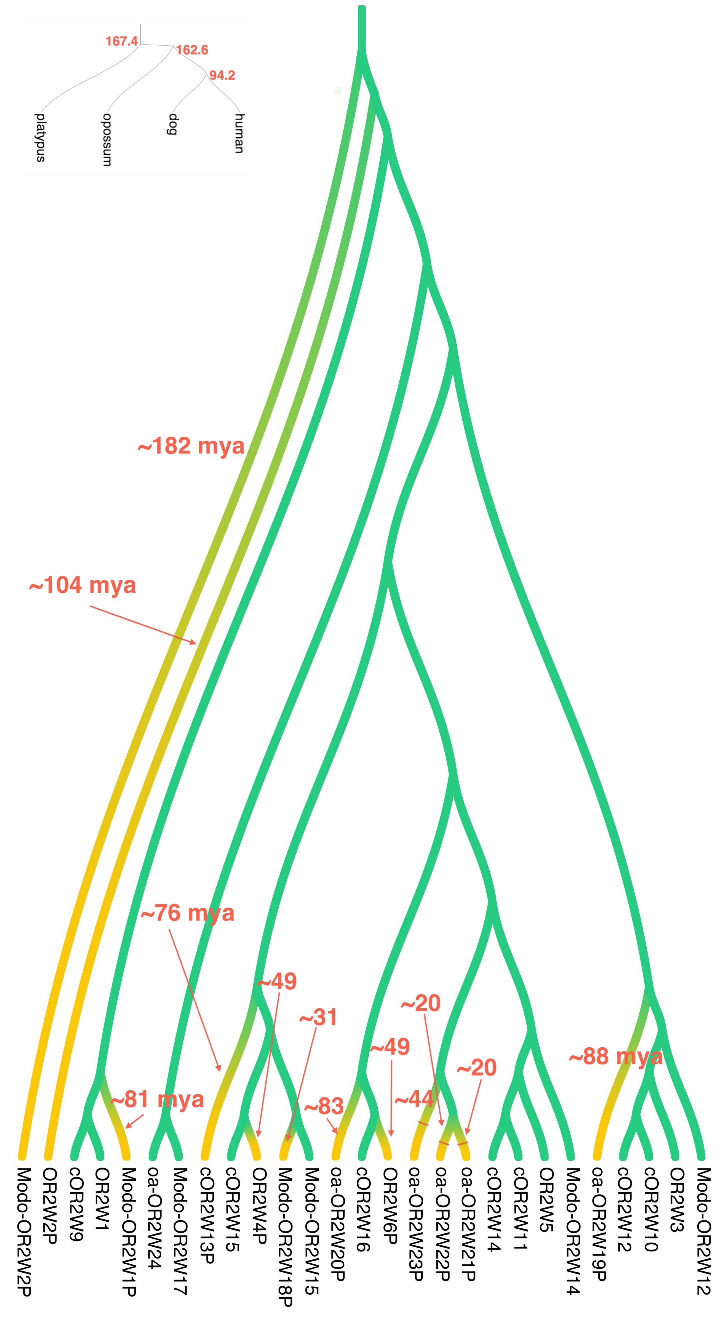

Figure S4: OR2W (Olfactory Receptors)

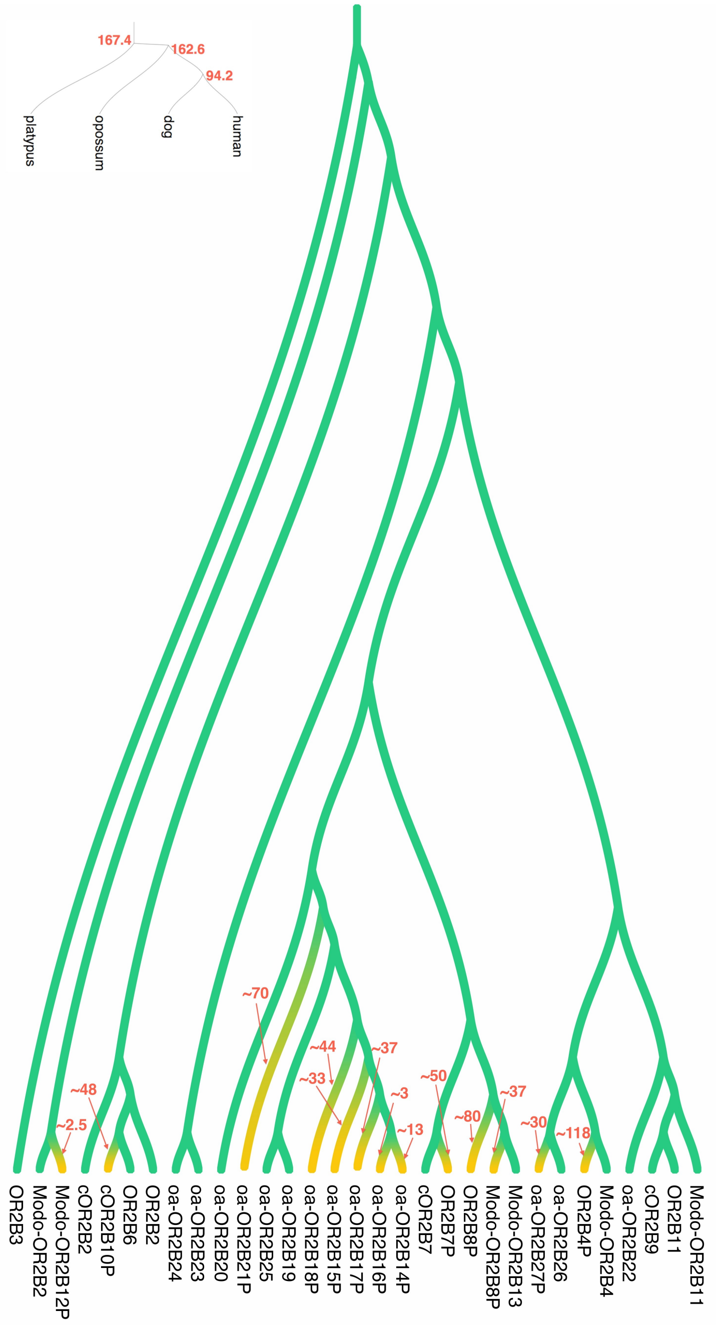

Figure S5: OR2B (Olfactory Receptors)

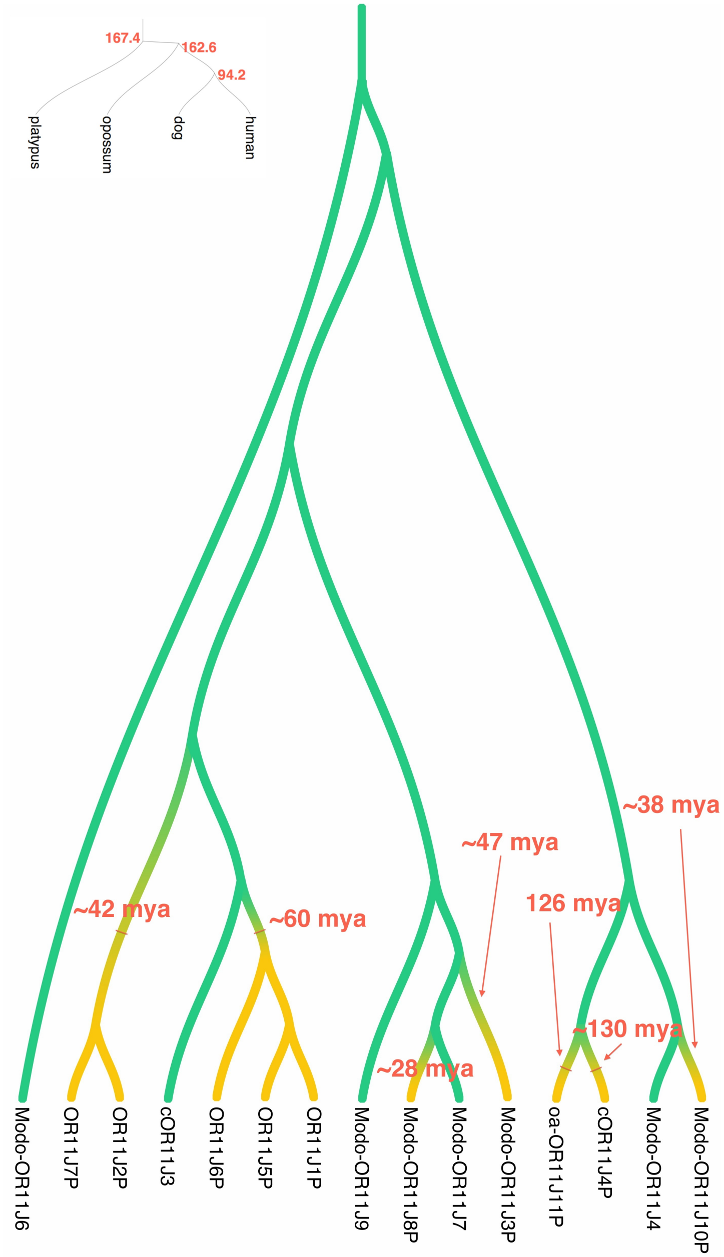

Figure S6: OR11J (Olfactory Receptors)

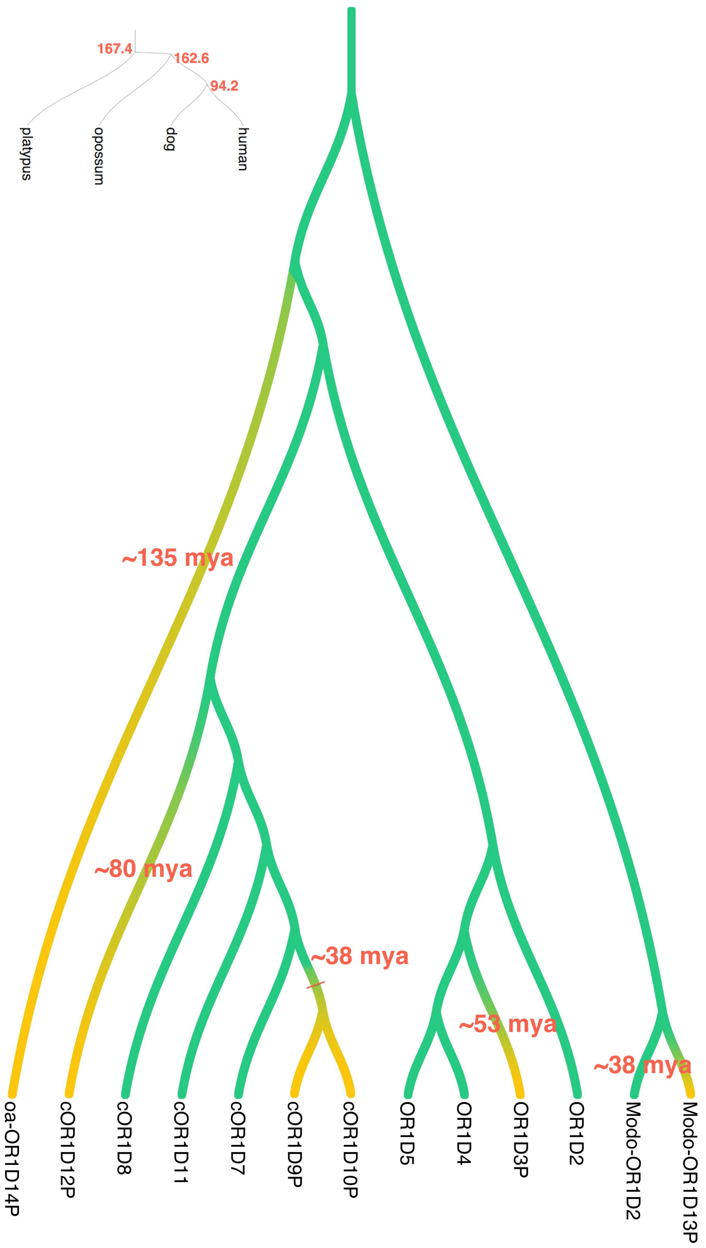

Figure S7: OR1D (Olfactory Receptors)

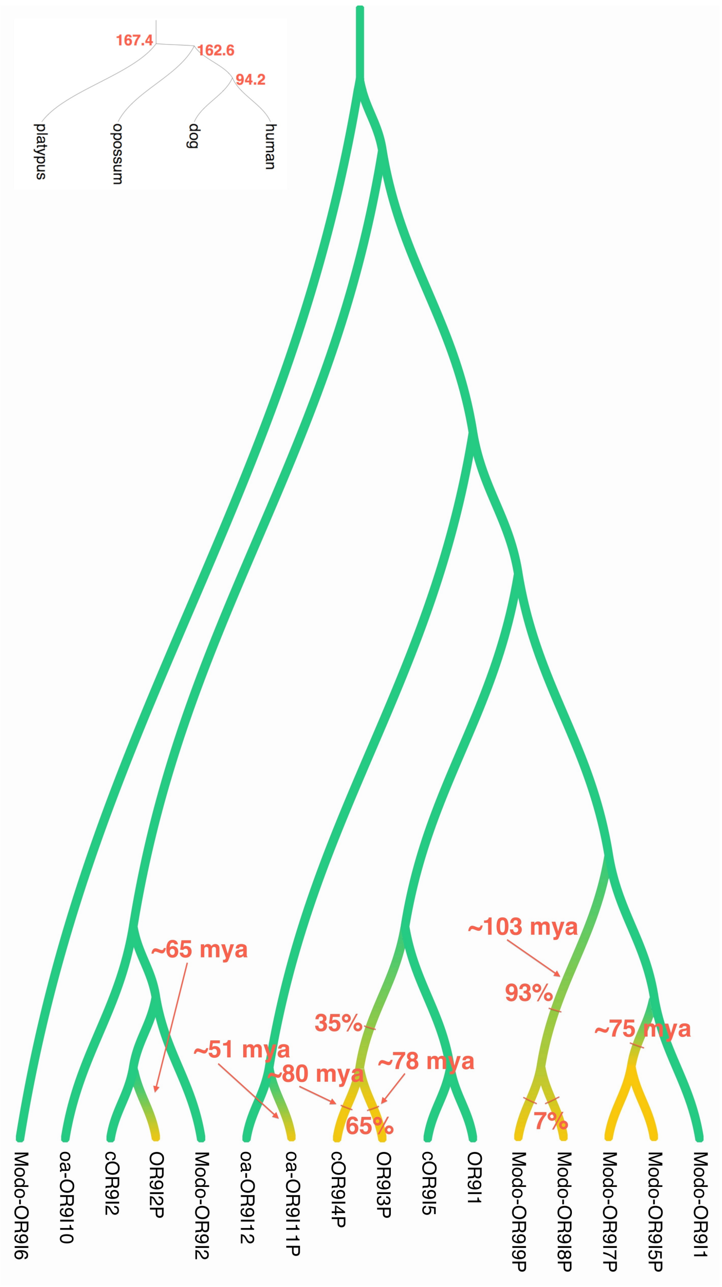

Figure S8: OR9I (Olfactory Receptors)

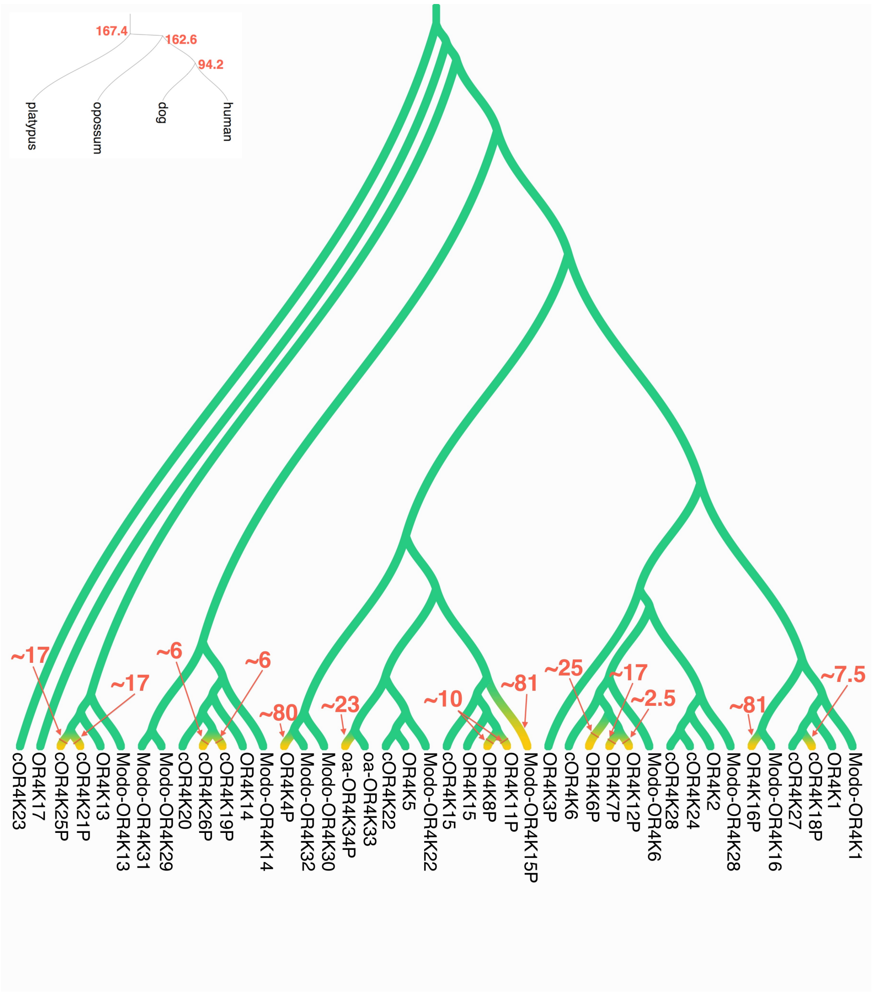

Figure S9: OR4K (Olfactory Receptors)
